# Determining the Influence of Habitual Dietary Protein Intake on Physiological Muscle Parameters in Youth and Older Age

**DOI:** 10.3390/nu13103560

**Published:** 2021-10-12

**Authors:** Sophie L. Mathewson, Adam L. Gordon, Kenneth Smith, Philip J. Atherton, Carolyn A. Greig, Bethan E. Phillips

**Affiliations:** 1 School of Sport, Exercise, and Rehabilitation Sciences, University of Birmingham, Edgbaston B15 2TT, UK; SLM545@student.bham.ac.uk (S.L.M.); C.A.Greig@bham.ac.uk (C.A.G.); 2 Medical Research Council-Versus Arthritis Centre for Musculoskeletal Ageing, University of Birmingham, Edgbaston B15 2TT, UK; 3 Medical Research Council-Versus Arthritis Centre for Musculoskeletal Ageing and NIHR Nottingham Biomedical Research Centre, University of Nottingham, Derby DE22 3DT, UK; Adam.Gordon@nottingham.ac.uk (A.L.G.); Ken.Smith@nottingham.ac.uk (K.S.); Philip.Atherton@nottingham.ac.uk (P.J.A.); 4 Department of Medicine for the Elderly, University Hospitals of Derby and Burton NHS Foundation Trust, Derby DE22 3NE, UK; 5 NIHR Birmingham Biomedical Research Centre, University Hospitals Birmingham NHS Foundation Trust and the University of Birmingham, Edgbaston B15 2TT, UK

**Keywords:** muscle protein synthesis, ageing, dietary protein, sarcopenia

## Abstract

Protein ingestion is a potent stimulator of skeletal muscle protein synthesis (MPS). However, older adults demonstrate resistance to anabolic stimuli. Some evidence has demonstrated that a larger acute protein dose is required in older compared to younger adults to elicit the same synthetic response, suggesting that older adults should be consuming higher habitual dietary protein to optimise muscle mass. However, limited research has explored dietary habits in different age groups or the relationship between habitual dietary intake and mechanistic physiological parameters associated with muscle mass and function. This work investigated the effect of habitual dietary intake in young (*n* = 10, 25.9 (3.2y)) and older (*n* = 16, 70.2 (3.2y)) community-dwelling adults (16:10 male: female) on physiological muscle parameters. Dietary intake was assessed using four-day diet diaries. Post-absorptive MPS and MPS responses to feeding (4.25x basal metabolic rate; 16% protein) were determined in muscle biopsies of the *m. vastus lateralis* via stable isotope tracer ([1, 2^−13^C_2_]-leucine) infusions with mass-spectrometric analyses. Body composition was measured by dual-energy x-ray absorptiometry. Whole body strength was assessed via 1-repetition maximum assessments. No significant differences in habitual dietary intake (protein, fat, carbohydrate and leucine as g.kgWBLM^−1^.day^−1^) were observed between age groups. Whole-body lean mass (61.8 ± 9.9 vs. 49.8 ± 11.9 kg, *p* = 0.01) and knee-extensor strength (87.7 ± 28.3 vs. 56.8 ± 16.4 kg, *p* = 0.002) were significantly higher in young adults. Habitual protein intake (g.kg^−1^.day^−1^) was not associated with whole-body lean mass, upper-leg lean mass, whole-body strength, knee-extensor strength, basal MPS or fed-state MPS across both age groups. These findings suggest that differences in muscle mass and strength parameters between youth and older age are not explained by differences in habitual dietary protein intake. Further research with a larger sample size is needed to fully explore these relationships and inform on interventions to mitigate sarcopenia development.

## 1. Introduction

We are currently living with an ageing population, with the number of older adults aged over 65 expected to accelerate from ~542 million in 2010 to nearly 1.5 billion in 2050 [1]. Increasing age is associated with a decline in muscle mass and function. This decline is termed sarcopenia, and is a known multifactorial condition associated with loss of independence [2], increased risk of falls and fractures [3], and increased morbidity and mortality [4]. In addition to losses of muscle mass and function, and recognised in the latest consensus on sarcopenia definition and diagnosis from the European Working Group on Sarcopenia in Older People (EWGSOP) [5], declines in muscle quality are also implicated in the development of sarcopenia, particularly in influencing muscle function [6]. Muscle quality can be defined as muscle strength per unit muscle mass, and is contributed to by many factors including intramuscular fat infiltration, neural activation, and muscle composition (muscle fibre type, cross-sectional area, and muscle thickness) [7]. Given that contractile activity (i.e., exercise, and particularly resistance exercise [8]) and essential amino acids (EAAs; [8,9]) are accepted as the two most potent anabolic stimuli, sarcopenia is likely aggravated by reductions in physical activity and/or lower dietary protein intake, both of which are reported with advancing age [10], and each of which have been shown to be associated with low muscle mass in older adults [11,12]. In addition, it has been demonstrated that the muscle of older adults is not able to robustly increase skeletal muscle protein synthesis (MPS) in response to these key anabolic stimuli (i.e., resistance exercise [13] and hyperaminoacidemia [6,14]) when compared to younger individuals. This phenomenon has been termed anabolic resistance [6] and has been postulated to be a significant contributor to the development and progression of sarcopenia [15].

Acute protein ingestion has been shown to result in acute increases in MPS, resulting in a positive net protein balance and promoting muscle mass maintenance and growth [8]. Despite the importance of protein ingestion as a robust acute stimulator of MPS via the provision of EAAs [16], increasing evidence demonstrates that older individuals consume less dietary protein compared to younger adults [17]. Indeed, despite observational and interventional evidence showing that higher protein intake elicits increases in muscle mass and strength [18,19,20,21] and quality of life [22], many older adults do not consume the daily recommended allowance (RDA) of protein (0.8 g.kg^−1^.day^−1^) [23]. Furthermore, despite evidence supporting the beneficial effect of habitual protein intake on muscle size and strength, its impact on mechanistic parameters underlying these benefits (i.e., MPS), is not widely reported. For example, higher daily protein intake has been reported to be associated with a preservation of muscle mass and increased quality of life in community-dwelling older adults [24,25], and increasing daily protein intake (from 1.2 to 1.6 g g.kg^−1^.day^−1^) has been shown to improve both whole-body [20] and appendicular lean mass [21] in older adults. However, these studies are interventional in nature and as such investigate the influence of protein supplementation over a period of time, as opposed to investigating the impact of true habitual protein intake.

Based on the concept of anabolic resistance [15], some evidence indicates that older individuals need to consume a higher acute protein dose to elicit the same MPS feeding response as younger adults [26]. This suggests that in order to minimise sarcopenia development, older adults should be consuming more protein in their habitual daily diet to maximise their cumulative MPS responses. However, the relationship between habitual protein intake and MPS has not been fully investigated, and therefore it is unclear whether higher habitual protein intake will impact fasted MPS rates, and/or MPS responses to feeding in older adults. Therefore, this study aimed to investigate differences in the habitual diets of independent community-dwelling, healthy young and older adults who were studied prior to participation in an exercise training intervention [27], and explore the relationship between habitual protein intake and muscle mass, function and protein metabolism (i.e., MPS).

## 2. Materials and Methods

The presented data are a secondary analysis of previously published data [27]. Briefly, young (*n* = 11, 25 ± 4 y, BMI: 23.3 ± 2.5 kg/m^2^) and older (non-sarcopenic, *n* = 16, 70 ± 3 y, BMI: 26.8 ± 2.0 kg/m^2^) participants were recruited through the local community. Potential participants underwent health screening via a medical questionnaire, physical examination, and ECG. Exclusion criteria included comorbidities and signs of ill health, participation in a regular (2 or more sessions of structured physical activity per week) exercise regimen and taking any form of nutritional supplementation (full exclusion criteria available in previously published work [27]). All participants provided informed written consent, and all screening and study procedures took place at the University of Nottingham Medical School at the Royal Derby Hospital Centre. All procedures were conducted in accordance with the Declaration of Helsinki and were given favourable opinion by the University of Nottingham Faculty of Medicine and Health Sciences Research Ethics Committee. Knee-extensor strength (KES; 1 repetition maximum (1-RM)) was assessed using a free-standing resistance exercise training machine (Leisure Lines, Hinckley, Leicestershire UK), with whole-body strength (WBS) determined as the sum of 1-RMs produced by 3 lower- (leg extension, leg curl, leg press) and 3 upper-body exercises (latissimus pull down, lever seated row, seated chest press).

Following confirmation of eligibility, participants attended after an overnight fast (water ad libitum) for an acute study visit. At this visit, body composition (including an upper-leg region of interest (ROI) given the functional importance of the muscle groups in this region [28]) was measured using dual-energy X-ray absorptiometry (DXA; Lunar Prodigy II, GE Medical Systems). A primed, constant infusion of [1, 2^−13^C_2_] leucine tracer (0.66 mg/kg, 1 mg/kg/h, 99 atoms percent; Cambridge Isotopes Ltd.) was then continually infused throughout the duration of the study (250 min). The tracer dose was increased (to 1.2 mg/kg/h) upon nutrition provision (at 130 min) to prevent tracer dilution. At 0, 120, and 250 min, using 1% lidocaine (B. Braun Melsungen) as local anaesthetic, *m. vastus lateralis* muscle biopsies were collected using the conchotome biopsy technique [29]. Muscle samples were prepared accordingly and stored at −80 °C until further analysis. After the second biopsy (~130 min) participants received an oral feed (Fortisip, Nutricia Clinical Care) with composition similar to that of a normal mixed meal (16% protein, 49% carbohydrate, 35% fat). This feed was provided as an initial bolus (three doses), followed by four further doses at 30-min intervals thereafter. Doses were between 61 and 96 mL based on body weight to provide 6.5 kJ/kgBW/30 min [27].

Prior to the acute study, all participants maintained a 4-day diet diary of all food and drink, with calibrated scales provided to each participant to facilitate this [30]. These diaries were analysed using Microdiet software v5 (Downlee Systems Ltd., High Peak, Derbyshire, UK).

Statistical analysis was performed using GraphPad Prism v.9.0.0. All data are reported as mean ± SEM, with significance set at *p* < 0.05. Unpaired *t*-tests were used to determine between group differences in habitual dietary intake and muscle-centric parameters between young and older adults. Linear regression and Pearson’s correlation analysis were used to explore relationships between habitual protein intake and muscle mass, function, and protein synthesis.

## 3. Results

**Figure 1:**
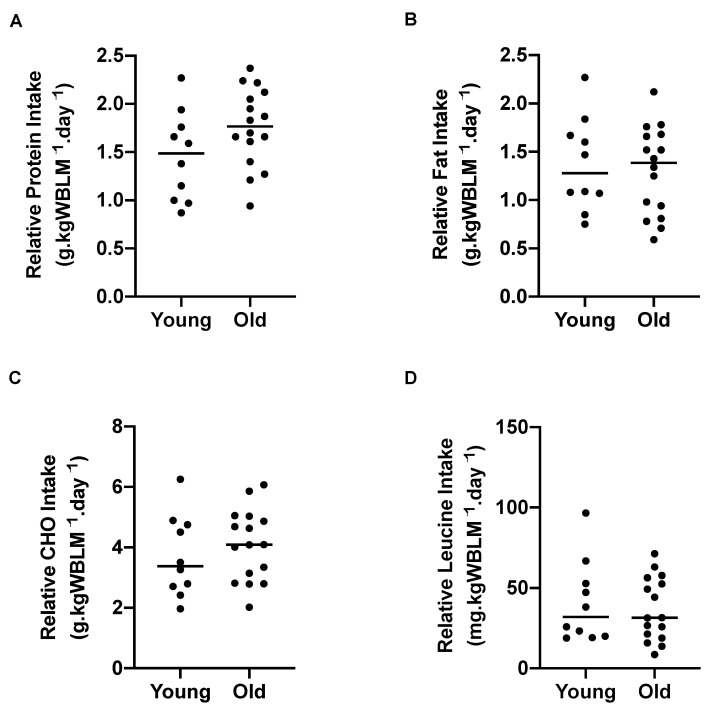
Habitual dietary intake parameters in young (*n* = 10) and older (*n* = 16) adults. (**A**) Protein intake (*p* = 0.62), (**B**) fat intake (*p* = 0.81), (**C**) carbohydrate (CHO) intake (*p* = 0.62), and (**D**) leucine intake (*p* = 0.35). Analysis via unpaired *t*-tests.

There was no significant difference in relative protein (Figure 1A), fat (Figure 1B), carbohydrate (Figure 1C) or leucine (Figure 1D) intake between young and older adults when expressed relative to whole body lean mass (g or mg.kgWBLM^−1^.day^−1^). No significant differences were observed in relative protein, carbohydrate, or leucine intake between young and older adults when expressed relative to body weight (g or mg.kgBW^−1^.day^−1^ (Table S1)). However, a significant difference was observed in relative fat intake between young and older adults (*p* = 0.01) when expressed relative to BW.

**Figure 2:**
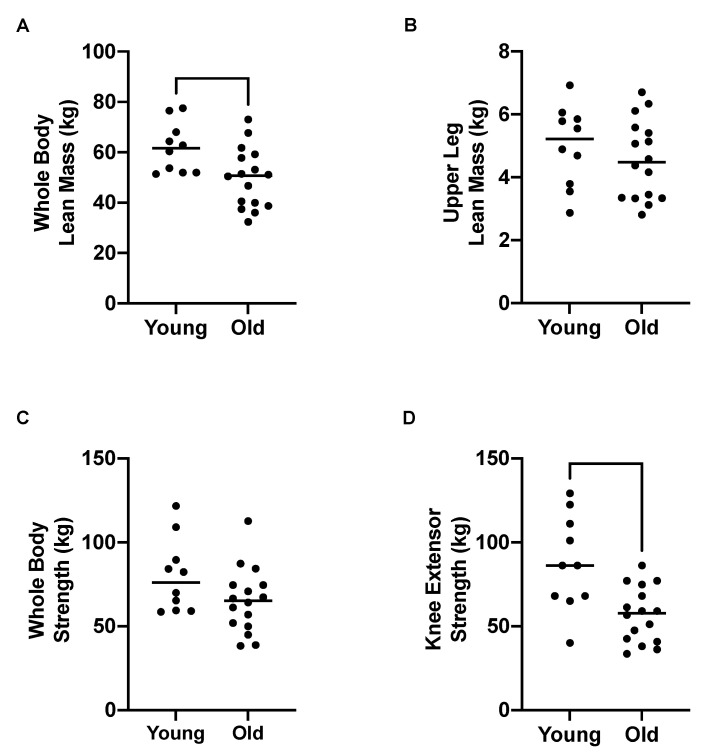
Muscle mass and strength parameters in young (*n* = 10) and older (*n* = 16) adults. (**A**) Whole body lean mass, (**B**) upper leg lean mass, (**C**) whole body strength, and (**D**) knee extensor strength. Analysis via unpaired *t*-tests.

WBLM was significantly higher in young compared to older adults (Figure 2A, 61.8 ± 9.9 vs. 49.8 ± 11.9 kg, *p* = 0.01), although there was no significant difference in upper lean leg mass (ULLM) (Figure 2B; 4.99 ± 1.28 vs. 4.55 ± 1.25 kg, *p* = 0.40). Despite this lack of difference in ULLM (selected as inguinal crease to mid-point of the patella), young adults demonstrated significant KES compared to older adults (Figure 1D, 87.7 ± 28.3 vs. 56.8 ± 16.4 kg, *p* = 0.002), although WBS was not significantly different (Figure 2C; 79.9 ± 21.9 vs. 65.3 ± 19.4 kg, *p* = 0.08) between the age groups.

**Figure 3:**
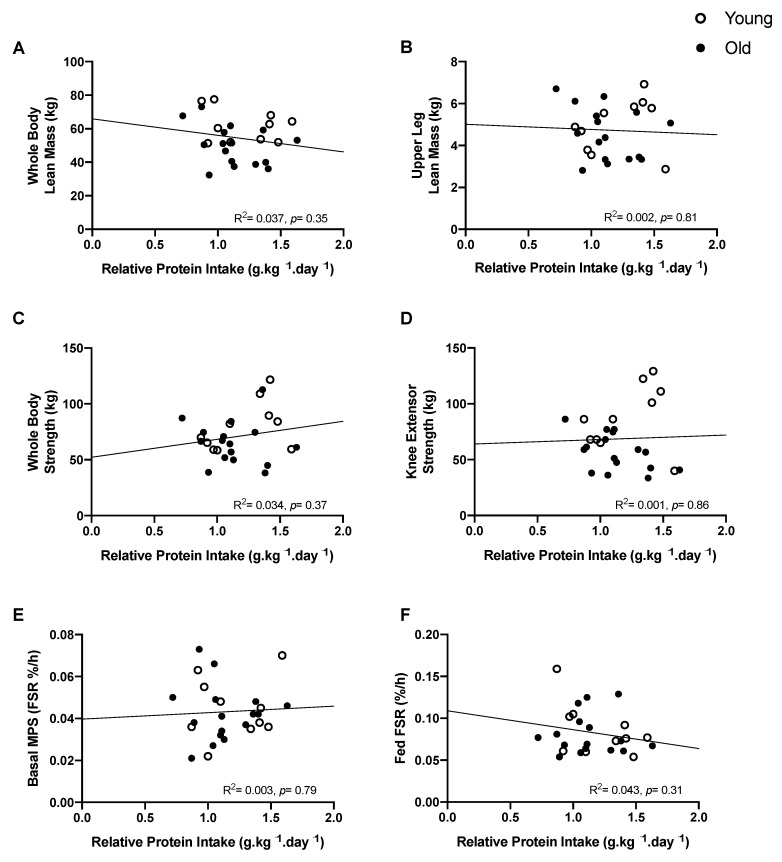
The relationship between relative protein intake expressed relative to body weight and physiological muscle parameters in young (*n* = 10; open circles) and older (*n* = 16; open circles) adults. (**A**) Whole body lean mass, (**B**) upper leg lean mass, (**C**) whole body strength, (**D**) knee extensor strength, (**E**) basal MPS, (**F**) fed state MPS. Analysis via linear regression on the combined groups.

As there was no significant difference in relative protein intake (g.kgWBLM^−1^.day^−1^ or g.kgBW^−1^.day^−1^) between young and older adults, data from both age groups were pooled to explore the relationship between facets of habitual protein intake and muscle mass, and metabolic and functional parameters. There was no significant relationship between habitual protein intake (in g.kg^−1^.day^−1^) and WBLM (R^2^ = 0.037, *p* = 0.35) or ULLM (R^2^ = 0.002, *p* = 0.81) (Figure 3A,B)). There was also no relationship with muscle strength (WBS (R^2^ = 0.034, *p* = 0.37) or KES (R^2^ = 0.001, *p* = 0.86)) (Figure 3C,D)) or MPS, either in the basal state (R^2^ = 0.003, *p* = 0.79) nor in response to feeding (R^2^ = 0.043, *p* = 0.31) (Figure 3E,F). There was also no relationship with any aspect of muscle mass, function, or metabolism when protein intake was expressed relative to WBLM (ULLM: R^2^ = 0.029, *p* = 0.40; WBS: R^2^ = <0.01, *p* = 0.91; KES: R^2^ = 0.022, *p* = 0.47; basal MPS: R^2^ = <0.001, *p* = 0.98; postprandial MPS: R^2^ = 0.116, *p* = 0.09). Similarly, there was no relationship between habitual leucine intake relative to either BW (WBLM: R^2^ = 0.013, *p* = 0.57; ULLM: R^2^ = 0.024, *p* = 0.45; WBS: R^2^ = 0.014, *p* = 0.57; KES: R^2^ = 0.002, *p* = 0.83; basal MPS: R^2^ = 0.029, *p* = 0.40; postprandial MPS: R^2^ = 0.006, *p* = 0.71) or WBLM and our measures of muscle mass, function and metabolism (WBLM: R^2^ = 0.109, *p* = 0.10; ULLM: R^2^ = 0.019, *p* = 0.50; WBS: R^2^ = 0.011, *p* = 0.62; KES: R^2^ = <0.01, *p* = 0.99; basal MPS: R^2^ = 0.055, *p* = 0.25; postprandial MPS: R^2^ = 0.027, *p* = 0.42).

## 4. Discussion

Herein we found no significant differences in habitual dietary intake expressed relative to either WBLM or BW between young and older adults, and no relationship between indices of habitual protein intake and measures of muscle mass, function or MPS. Further, we observed no significant difference in ULLM between healthy community-dwelling young and older adults but do report significantly lower KES in older adults. These findings support the notion of declines in muscle function, perhaps indicative of reduced muscle quality [31], preceding a decline in muscle mass.

The current RDA for protein intake in both young and older adults is 0.8 g.kg^−1^.day^−1^ [32], however, there are suggestions that this should be higher in older adults [33], especially in individuals with chronic disease or injury or individuals experiencing severe malnutrition [19]. When considering these guidelines, we observed that all the young adults and all the older adults in this study except one (94%) achieved this threshold. Further, even when considering the suggestion of alternative guidelines for healthy older adults as proposed by the PROT-AGE group of 1.0–1.2 g.kg^−1^.day^−1^ [19], only 25% of the older adults in this study did not achieve this. These results highlight that, based on the individuals recruited to this study, independent, community-dwelling, healthy older adults are consuming the recommended amount of protein to aid skeletal muscle maintenance and growth. This is in contrast to previously published results investigating the dietary habits of young, middle-aged, and older adults, which reported a significant difference in relative protein intake between young and older adults [17], and also reported that 65% of older adults did not reach a protein intake of 1.0 g.kg^−1^.day^−1^. However, Smeuninx et al., did report on a larger sample (*n* = 40 young and *n* = 40 older adults) and importantly recruited an older age group that was on average 7 years older than in this current study. Aside from protein intake, another important dietary factor to consider in influencing skeletal muscle health is EAA intake [9], particularly leucine [34]. There is a growing body of evidence supporting the positive role of protein quality on skeletal muscle health, with affirmation that the capacity of a protein source to stimulate MPS is due, at least in part, to the leucine content of the protein [34,35,36]. We report no differences in habitual leucine intake between young and older adults, in addition to no relationship between relative leucine intake and any muscle composition, function, or MPS. These results suggest that neither protein quality nor insufficiency is likely to explain the reductions in muscle mass and function (in the knee-extensors, a muscle group crucial for activities of daily living [28]) that we observed.

Although sarcopenia is an umbrella term used to describe whole-body declines in muscle mass and function [5], recent research has demonstrated specific early losses of lower limb mass and function with advancing age in both men and women [37,38,39,40], supported by our findings of age-associated reductions in KES but not WBS. This finding is similar to that of previous longitudinal studies demonstrating losses of KES over 3, 9, and 10 [31,40,41] in older adults. However, contrary to these suggestions of early losses of lower limb mass, our results displayed no significant differences in ULLM between young and older adults, although WBLM was lower in older age. The decreases in lower limb muscle strength that we observe in older adults, even in the absence of reductions in lean mass, may be due in part to decreases in muscle quality such as muscle architecture [42], myosteatosis [43] and/or neuromuscular function [44], each of which have been shown to impact muscle function [31,42]. It must, however, also be considered that these results may reflect inherent limitations of DXA not being sufficiently sensitive to detect subtle differences in body composition [45,46], especially given the relatively small sample size and physiological heterogeneity (e.g., males and females) of our groups.

The anabolic effect of acute protein ingestion on skeletal muscle is well-reported, however, the impact of habitual protein intake on physiological muscle parameters supporting muscle mass and function, in particular MPS, has not been widely investigated. Previous research has reported that a lower acute protein dose is needed to maximally stimulate MPS in younger compared to older adults (0.24 g/kg vs. 0.40 g/kg) [26]. However, it is unclear whether these findings also apply when considering the habitual protein intake. A retrospective analysis of previously published work [16,35,47,48,49,50] suggested that healthy older men may have attenuated sensitivity to low protein intake [26], thereby requiring a higher protein intake relative to BW to maximally stimulate MPS. We observed no relationship between habitual relative protein intake and MPS in either the fasted or fed state. Although the role of relative habitual protein intake on MPS is not known, two-weeks of habituation to a low or high protein diet (0.7 g/kg vs. 1.5 g/kg) did impact amino acid (AA) availability. This augmentation was likely due to reductions in splanchnic AA retention in the low protein group [51]. However, despite this, there were no differences in MPS responses to an acute dose of whey protein (25 g), possibly due to a redistribution of protein-derived AAs to other tissues [51]. When interpreting the presented results, it is important to consider the influence of pre-sleep protein and fasting on MPS levels following an acute protein dose. MPS levels are suppressed overnight due to limited AA availability, however, some research suggests that ingestion of pre-sleep protein (~40 g) results in elevated MPS overnight, particularly following exercise training [52]. In the present study, participants attended the laboratory following an overnight fast. Overnight fasting (~10 h) has been demonstrated to increase MPB and decrease AA oxidation levels, suggestive that a larger acute protein bolus may be needed to maximise MPS levels following an overnight fast [53]. However, further research is needed to fully determine the influence of habitual protein intake on MPS responses.

This work is not without limitations. The older adults in the present study were community-dwelling older volunteers, and as such were relatively healthy, high functioning and were not classed as sarcopenic. Future research needs to include a wider range of older adults, including those with sarcopenia, and potentially those in residential care facilities who represent a growing proportion of older adults [54], and who are known to present with distinct characteristics (e.g., dysphagia [55], polypharmacy [54]) with potential to impact nutrient intake and absorption **[56]**. Studying protein metabolism in such populations is not without challenges, but the development of new oral tracer techniques such as those using D_2_O which can measure MPS in longer-term ‘free-living’ scenarios may provide additional insight into the impact of habitual protein intake on muscle mass and function in these ‘at-risk’ populations [57,58]. In addition, given inter-individual variation in dietary habits due to lifestyle constraints (i.e., cost) and food preferences across all ages, a larger sample size is needed to confirm the findings of this work.

## 5. Conclusions

In conclusion, the findings of this work suggest that high functioning, community-dwelling, healthy older adults are consuming the recommended daily amount of protein, and that their dietary intake is not different to that of younger adults. As such, these findings suggest that the age-associated reductions in muscle mass and function observed in this study are not attributable to low protein intake. The lack of relationship between habitual protein intake and MPS highlights the role that other factors, such as physical activity, likely play in maintaining skeletal muscle mass and function. Further research with a larger sample size and more diverse ageing populations (i.e., older adults including those with comorbidities and/or supported living environments) is needed to fully investigate this relationship.

## Data Availability

The data used in the presented secondary data analysis is from previously published work [27].

## Supplementary Materials

The following are available online at https://www.mdpi.com/article/10.3390/nu13103560/s1. Table S1: Differences in dietary intake between young and older adults. Abbreviations: WBLM, whole-body lean mass; BW, body weight. N = 11 young and N – 16 older adults. * represents a statistically significant difference compared to younger adults (*p* < 0.05). All data presented as mean ± SEM.
