# Supplementary material for: Determining the Influence of Habitual Dietary Protein Intake on Physiological Muscle Parameters in Youth and Older Age
Source: Nutrients. 2021 Oct 12;13(10):3560. doi: 10.3390/nu13103560 (PMC8539198; doi:10.3390/nu13103560)
Supplement: Supplementary file 1 [file nutrients-13-03560-s001.zip › nutrients-1384954-supplementary.pdf]

nutrients

Supplementary Materials

Table S1. Differences in dietary intake between young and older adults

	Young Per kg WBLM	Older Per kg WBLM	Young Per kg BW	Older Per kg BW
Protein (g)	1.46 ± 0.15	1.76 ± 0.10	1.21 ± 0.10	1.13 ± 0.06
Fat (g)	1.37 ± 0.15	1.30 ± 0.11	1.14 ± 0.10	0.83 ± 0.06*
Carbohydrate (g)	3.71 ± 0.43	4.08 ± 0.29	3.04 ± 0.22	2.62 ± 0.17
Leucine (mg)	40.84 ± 8.09	36.77 ± 4.90	33.33 ± 5.30	23.57 ± 11.89

Abbreviations: WBLM, whole-body lean mass; BW, body weight. N = 11 young and N – 16 older adults. * represents a statistically significant difference compared to younger adults (*P* < .05). All data presented as mean ± SEM.
